# Social interaction in augmented reality

**DOI:** 10.1371/journal.pone.0216290

**Published:** 2019-05-14

**Authors:** Mark Roman Miller, Hanseul Jun, Fernanda Herrera, Jacob Yu Villa, Greg Welch, Jeremy N. Bailenson

**Affiliations:** 1 Department of Computer Science, Stanford University, Stanford, CA, United States of America; 2 Department of Communication, Stanford University, Stanford, CA, United States of America; 3 Department of Computer Science, University of Central Florida, Orlando, FL, United States of America; Birkbeck University of London, UNITED KINGDOM

## Abstract

There have been decades of research on the usability and educational value of augmented reality. However, less is known about how augmented reality affects social interactions. The current paper presents three studies that test the social psychological effects of augmented reality. Study 1 examined participants’ task performance in the presence of embodied agents and replicated the typical pattern of social facilitation and inhibition. Participants performed a simple task better, but a hard task worse, in the presence of an agent compared to when participants complete the tasks alone. Study 2 examined nonverbal behavior. Participants met an agent sitting in one of two chairs and were asked to choose one of the chairs to sit on. Participants wearing the headset never sat directly on the agent when given the choice of two seats, and while approaching, most of the participants chose the rotation direction to avoid turning their heads away from the agent. A separate group of participants chose a seat after removing the augmented reality headset, and the majority still avoided the seat previously occupied by the agent. Study 3 examined the social costs of using an augmented reality headset with others who are not using a headset. Participants talked in dyads, and augmented reality users reported less social connection to their partner compared to those not using augmented reality. Overall, these studies provide evidence suggesting that task performance, nonverbal behavior, and social connectedness are significantly affected by the presence or absence of virtual content.

## Introduction

Augmented reality (AR) has captured the attention of both the public and corporations with its ability to seamlessly integrate digital content with the real-world environment. AR devices like the Microsoft HoloLens and Magic Leap One allow users to see the real physical world but superimpose a layer of digital content such that users see virtual models mixed in with the actual world around them. Typically, the digital objects–which can be anything ranging from a simple shape to a realistic model of a person–are rendered in stereo (i.e., with separate images projected into each eye) to give the illusion of depth when situated next to real objects. Moreover, the digital objects are *registered*, using cameras and sensors that track a user’s position in an absolute location, such that when a person moves, the object stays in the programmed position.

While the general interest in AR may be recent and growing, academic researchers have been building and testing the technology for decades [1,2]. Many experiments focus on the technical and design aspects of AR, but less is known about how *social interactions* are affected by the technology. Gordon Allport [3] defined the field of social psychology as “an attempt to understand and explain how the thoughts, feelings, and behavior of individuals are influenced by the actual, imagined, or implied presence of others”. This well-accepted definition is broad enough to include virtual people rendered in AR. Allport’s foresight in extending social psychology to imagined and implied others is quite prescient, given the new types of social interactions which will become possible in AR.

Imagine a cocktail party, with dozens of people socializing, about half of whom are wearing AR headsets. Perhaps some people will choose to invite other virtual avatars into the party, and these can only be seen by those wearing headsets. The presence of avatars might change the way people talk, gesture, and socialize. For example, what happens when an AR user at the party is forced to violate the personal space of either an avatar who is registered in a specific location or a real human who is not aware of the avatar? While similar issues occur with phone calls and videoconferencing, the unique aspect of AR is that these avatars are grounded spatially in the room in a set position among the physical people.

In the example above, the people rendered into the cocktail party are *avatars*, a real-time rendering of other people. Collaborative AR systems, like the one in the cocktail party example, allow remote or co-located users to interact with each other. Co-located systems usually consist of two or more users wearing AR headsets in the same physical location and are able to see and interact with the same virtual content. Remote systems usually consist of two or more users in different physical locations. A common example consists of one user wearing a headset while remote others assist in a specific task by looking at the same content through either a monitor or a second AR headset [4].

Collaborative AR systems have been explored as tools to improve completion time and reduce mental effort in design tasks [5,6], facilitate communication between collaborators [7] and increase mutual understanding [8]. For a review, see [9]. Overall, these pioneering studies focused on testing the efficacy of various systems, usability, and design, but have yet to examine the social psychological aspects of AR.

Other AR systems feature social interaction by employing *embodied agents*, which are characters whose verbal and nonverbal behavior are generated algorithmically in response to users’ behavior. For example, in Fragments [10] the application which as of May 2019 has received more user ratings than any other application for the Microsoft HoloLens [11], the AR user interacts with many embodied agents while solving a crime in a game that integrates digital and physical objects into the game’s narrative.

In immersive virtual reality (VR), there have been hundreds of papers examining how users respond to virtual humans, whether they are avatars or agents. For a recent review of this literature, see [12]. However, the extent to which these findings extend to virtual humans in AR is not yet known outside of a handful of studies. A recent review by Kim and colleagues [13] surveyed every paper published in the International Symposium on Mixed and Augmented Reality (ISMAR), the leading academic conference on AR, from the past decade (2008–2017). In their analysis, Kim and colleagues found an increasing trend in evaluative studies (i.e., studies in which AR systems are built and tested for their effectiveness or the psychological effects they have on users). For example, Steptoe, Julier, and Steed [14] demonstrated the difference in visual quality between rendered and real objects reduces feelings of presence. However, this decrease in presence disappeared when both the real and virtual objects were passed through an image filter, eliminating the difference in visual quality. According to the findings from the Kim and colleagues’ survey of ISMAR [13], less than two percent of all papers (9 of 526) examined any type of social collaboration.

Many of these studies in the subset of AR social interaction have focused on qualitative findings, or anecdotal accounts of how the system was received by users. For example, one AR interface presented participants with an agent that floated over their heads. Researchers noted the participants felt uncomfortable when it looked down at them [15]. Similarly, Wagner [16] created a conversational agent describing art pieces in a gallery. Some participants noted they felt insulted when the agent violated social norms and faced away from them while speaking to them. These qualitative observations suggest agents tend to elicit social responses and inform our understanding of human interactions with agents. However, more empirical evidence providing support for these claims is needed.

A number of studies by Welch and colleagues demonstrate that embodied agents can achieve higher social presence (as measured by self-report or behavioral data) by successfully integrating virtual content with the real world. In a study by Kim and colleagues [17], participants interacted with a virtual agent while a real fan was blowing in the room. Participants encountered one of three conditions: the fan did not affect the virtual world, the fan blew a virtual paper, or the virtual agent reacted to this airflow and held down the corner of the paper. The perceived social presence of the virtual agent was greater in the two conditions where virtual content reacted to real physical events. This work suggests that the more virtual content reacts to the physical environment, the more virtual humans seem real.

In another study by Kim and colleagues [18], a virtual agent in a virtual motorized wheelchair interviewed participants. In the two conditions, the virtual agent's behavior was either behaviorally unrealistic (e.g., it passed through doors, did not avoid physical objects, and was not occluded by physical objects in front of it) or is behaviorally realistic (e.g., requested to open the door, asked the participant to move a chair, and was occluded by physical objects in front of it). The participants who saw the behaviorally realistic agent had higher social presence scores and thought of the agent as more intelligent than participants who interacted with the agent that behaved unrealistically.

Moreover, embodiment can also increase social presence and trust in an agent performing tasks. A recent study had participants interact with an intelligent virtual assistant inspired by Amazon's Alexa. The embodiment of the virtual agent was either not visible, visible with gestures, or visible with gestures and locomotion (i.e., it was able to move across the room). Participants rated the agent with the highest level of fidelity (i.e., visibility, gestures, and locomotion) as more socially present and trustworthy compared to the other conditions [19]. This study suggests AR users prefer high fidelity agents that interact with objects in a room using socially typical animations rather than disembodied agents.

The present investigation uses the work by Welch and colleagues as a baseline, and focuses on new questions, namely how AR social interaction changes task performance, nonverbal behavior, and social connection with other physically co-located people. We review the relevant literature for each of these outcomes.

### Task performance: Social facilitation and inhibition

Much of the past work building and testing AR applications has been around task performance. In a review of AR applications [13], Kim et al. refer to six common types of applications: military, industry, healthcare, games, tours, and media. In the three that are most common (military, industry, and healthcare applications), the goal is often to improve task speed or quality [20–22].

The addition of virtual humans will likely influence performance. A well-studied theory in social psychology is social facilitation and inhibition. Social facilitation refers to the tendency of people to perform simple tasks faster in the presence of others. Conversely, social inhibition is the tendency to perform complex tasks poorly in the presence of others. These concepts were first studied 120 years ago when Triplett [23] noted faster winding speeds in a reel-winding competition for children in pairs when compared to children who completed the task alone. This effect was further investigated by Allport [24], who found people wrote free chain associations faster in the presence of others than alone, even when competition was explicitly barred. However, later research found an opposing effect. When participants tried to learn nonsense syllables in the presence of others rather than alone, they took more trials to memorize the syllables and made more errors during the process [25].

These contradictory effects were addressed by Zajonc’s drive theory [26], which was able to explain both enhanced and impaired performance in the presence of others by making a distinction between simple and complex tasks. Zajonc’s drive theory posits that the presence of others increases one’s arousal level, or drive, and that this increase in arousal leads to enhanced or impaired performance depending on task difficulty. Since then, multiple studies have replicated these social facilitation and inhibition findings (see [27] for a review). Other explanations have been given for the effect, including self-presentation theory (see [28] for a meta-analysis). Self-presentation theory posits that the audience strongly motivates the participant to perform well, which aids performance regardless of difficulty. The difference between easy and hard tasks arises when the performer becomes embarrassed or distracted by their poor performance on hard tasks.

It is important to note that the study of social facilitation and inhibition has not been limited to the physical presence of real people. Dashiell [29] evaluated performance of a mathematical task in two conditions. In each case, participants were physically alone in their individual experiment rooms; however, in one condition, subjects were aware that others were being tested at the same time. Even though participants had no contact with other participants, the implied presence of others was sufficient to elicit a social facilitation effect. More recently, researchers have expanded the study of social facilitation to include the presence of virtual others (i.e., avatars and agents) in immersive and non-immersive virtual environments.

Early research examining the effect of virtual humans consistently demonstrated a social inhibition effect, with participants struggling to perform complex tasks in front of virtual others, but had been unable to reproduce a social facilitation effect [30–32]. However, Park and Catrambone [33] were able to demonstrate both social facilitation and inhibition effects by using a different experimental method. Instead of having participants train on one task so the tasks varied in familiarity, as the earlier studies had done [30–32], participants were given tasks that were pretested to vary in difficulty. In this study, participants performed three tasks at two levels of difficulty (easy or hard) in the presence of a real person, the presence of an agent, or alone. Results showed both social facilitation and inhibition effects in both the virtual human and real human conditions.

In sum, there is a robust literature showing that an audience—both physically present and in immersive VR—influence performance. The extent to which this extends to AR is critical to understand, given that people may be using this technology in their daily lives, performing tasks ranging from navigation to repairs to business meetings.

### Nonverbal behavior: Interpersonal distance and eye-contact

Nonverbal behavior plays a major role in communication [34]. Two well-studied components of nonverbal communication are interpersonal distance and eye-contact. Interpersonal distance refers to the physical distance that individuals maintain during social interactions. Though there are many theoretical accounts of how people regulate interpersonal distance, in general, most people tend to choose a distance that is comfortable, given the context and social relationships among people (see [35] for a review). When it comes to eye-contact, past research has demonstrated that the use of eye-contact helps individuals regulate interactions, express intimacy, provide information, and facilitate collaboration [36].

Similarly, in immersive VR, one of the most studied aspects of nonverbal behavior is interpersonal distance and eye-contact (See [37] for a review). Early work demonstrated that users are reluctant to walk through other virtual humans [38], and that they tend to maintain interpersonal distance with virtual humans [39]. In the past 15 years, there have been many subsequent studies examining interpersonal distance between users and virtual humans in VR suggesting that users tend to follow this social norm with both avatars and agents (see [40] for a recent review). Additionally, past research has also demonstrated that users tend to maintain eye-contact with avatars and agents in virtual environments [41].

While previous work on interpersonal distance and eye-contact provides clear predictions for how people should interact with AR agents–by respecting their space and maintaining a proper distance and eye-contact during an interaction–predictions are less clear for what happens moments after they see the rendering of an agent. AR is different from most media in that it superimposes digital content onto the physical environment. Hence, this medium poses a novel consequence in the tradition of media effects in that users might form associations between the virtual content and the physical objects that may remain even after the AR headset is no longer in use and users are no longer able to see the virtual content. In other words, after the medium has been turned off, affective responses and behaviors towards specific physical objects might change due to the newly formed associations with previously rendered virtual content.

In sum, AR will present unique challenges to the norms of nonverbal behavior in which virtual humans are intermingled with physical ones. Given AR is designed to be used in public places around other physically located people, understanding the effects on nonverbal behavior are critical.

### Social connectedness: Users and non-users

In his 1992 novel, *Snow Crash*, Neal Stephenson discusses “Gargoyles,” people who use AR/VR in public. He explains that “Gargoyles are no fun to talk to. They never finish a sentence. They are adrift in a laser-drawn world” [42]. Of course, the same can be said for cell phone usage. Past research has demonstrated that the mere presence of smartphones during face-to-face (FtF) conversations has an effect on communication outcomes. More specifically, Przybylski and Weinstein [43] showed that when a smartphone was present during a conversation, partner closeness was lower despite the phone not playing an active part in the conversation. In a different study, Misra and colleagues [44] found that conversations between interaction partners in the absence of a smartphone were considered to be significantly higher in quality than conversations where there was a mobile device present. Additionally, people who had conversations in the absence of a smartphone had significantly higher empathic concern scores than people who had conversations in the presence of a smartphone. Vanden Abeele and colleagues [45] demonstrated that when one person actively uses their phone during conversation, their conversation partner formed more negative impressions of them, found them less polite, and possessed poorer perceptions of the quality of conversation compared to those that did not actively use their phone.

When it comes to see-through AR headsets specifically, one of the main goals is to superimpose virtual content onto the user’s real world environment with the purpose of providing the user with additional information about their surroundings [2]. This affordance makes it possible for AR users to interact with virtual content that is visible only to them, which may make bystanders curious or uncomfortable. Furthermore, virtual content may intentionally or unintentionally be rendered on top of people the AR user is interacting with, potentially disrupting the interaction by causing the violation of social norms. In the case of eye-contact, AR may create situations where establishing and maintaining eye-contact is difficult. For example, the AR headset itself may prevent users and non-users from making eye-contact. Additionally, virtual content that occludes people’s faces would lead to an inability for eye-contact to be established by the AR user. However, to our knowledge, there have been no studies assessing the effect of socially interacting with someone who is occluded by private, virtual content.

In AR, it is likely that other people who are not wearing an AR headset (i.e., non-users) in a room will not be aware of all the digital content being rendered to the users. The presence of AR objects or virtual humans may distract users, preventing them from focusing on the non-users they were interacting with. This may lead to a loss of *common ground*, or mutually shared information [46], between AR users and non-users, and to the violation of multiple social norms (e.g., eye-contact, turn-taking during a conversation, and interpersonal distance). A case study of a conversation between an AR user wearing Google Glass (a type of AR headset) and a non-user suggests that Glass disrupted turn taking between individuals, leading to poor rapport [47]. In the final pages of their survey on AR, Billinghurst, Clark, and Lee [1] focus on the challenges around social acceptance of AR. While Google Glass received some criticism, the authors point out there is still very little empirical research on how AR use is perceived by non-users and the effects that wearing the headset have on social interactions.

## Overview of studies

In this investigation, three studies were conducted to assess the social effects surrounding augmented reality use.

Study 1 examines how embodied agents affect the way people perform tasks in the physical world. A robust literature on social facilitation demonstrates that people tend to benefit from an audience when they perform easy tasks but tend to be impaired by the same audience when they perform difficult tasks. Given AR users will likely be rendering virtual humans in AR while they are performing daily tasks in the real world, the extent to which these processes are replicated in AR becomes an imperative question.

Study 2 examines how social interactions in AR will change users’ subsequent nonverbal behavior in the physical world. Given there is a spatial component to AR social interactions [48] it is likely that virtual humans in AR will be associated with objects or locations in the physical room where they were rendered. These associations may affect social behavior even after the virtual content is no longer displayed in the physical environment.

Study 3 examines the effect that the presence of see-through AR headsets during a FtF dyadic interaction has on interpersonal outcomes. Given virtual content is often rendered in specific locations in the physical environment, it is likely that some of it may partially or completely occlude people the AR user is interacting with. Thus, we also examine the effect that interacting with someone whose face is either completely occluded by virtual content or not occluded at all has on interpersonal outcomes.

## Study 1: Task performance

Given that the few experiments examining social interaction with AR agents have demonstrated that virtual humans in AR elicit social responses and affect communication outcomes [15–18], we hypothesize the following:

**Hypothesis 1.** *Compared to being alone*, *participants who perform an easy cognitive task in the presence of an AR agent will have enhanced performance while participants who perform a difficult cognitive task in the presence of the same AR agent will have impaired performance*.

### Methods

#### Participants

A total of 60 participants (32 female, 28 male) were recruited from Stanford University. The recruitment and experiment processes were approved by the Stanford IRB under protocol IRB-45211. The participants shown in figures have given written informed consent to publish their likeness under the CC-BY license. Participants were given course credit for completing the experiment. Fifty-eight participants’ ages were between 18 and 24, and two participants were between 25 and 35 years old. When choosing the sample size, we considered the closest work, which was [33]. Using an estimate of their effects, we ran a power analysis for a within-subjects design and found 60 subjects to be powerful at a β = 0.8 level. This also gave us 15 subjects in a between-subjects design, which is a typical sample size used in previous studies (see [49] for a list of sample sizes of similar studies).

#### Materials

**Cognitive Task.** The cognitive task completed by the participants was an anagram task. Similar to Park and Catrambone [33], anagrams were split into two groups, easy and hard. Easy anagrams were chosen from Tresselt and Mayzner’s [50] anagram set with median solution times between 3 and 13 seconds. Hard anagrams were chosen from the same set but had a median solution time between 17 and 143 seconds. From each of these sets, two posters of ten anagrams were selected, for a total of four posters. Within the same difficulty level, the anagrams were divided such that the posters would be as similar in difficulty as possible. For the complete list of anagrams see S1 Appendix.

**Fig 1:**
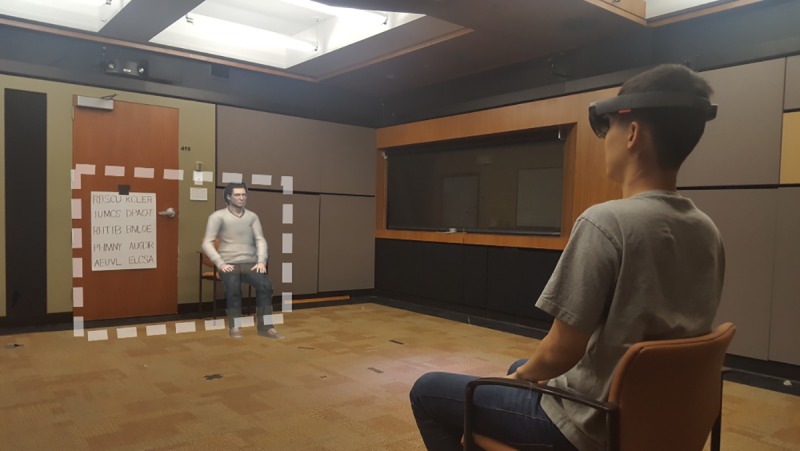
Over-the-shoulder view of an actor in place of a participant showing the anagram poster and the virtual human. The dashed area represents the field of view of the HoloLens.

**Apparatus.** Participants wore the Microsoft HoloLens AR headset with a 60 Hz refresh rate and a resolution of 1268 x 720 per eye. The virtual field of view of the HoloLens is about as large as a letter size or A4 paper at arm's length (i.e., horizontal and vertical fields of view of 30° and 17.5° respectively [51]). To see this size in the experiment space, refer to Fig 1. However, the field of view for physical objects was not impaired given the HoloLens is a see-through AR headset and participants can still see the physical environment around them. The device weighed 579 grams, recorded audio, and tracked headset position (x, y, z) and orientation (yaw, pitch, roll) [52].

The virtual environment was created as an application through the Unity game engine version 2017.3.1f1. The application also included a networking component connecting to the experimenter's laptop and the HoloLens. During the experiment, the researcher could change the virtual content displayed to the participant (e.g., make the agent present or absent). The participants’ solutions to the anagrams were recorded by the headset as an audio (.wav) file.

**Content.** The virtual agent, introduced as Chris, was matched to the participants’ biological sex in order to avoid sex effects. There was one model of each biological sex, and both male and female character models exhibited idling and walking animations. To simulate natural speech, the agent’s jaw bone was programmed to move up and down based on the volume of the recorded audio.

**Physical Space.** Participants sat on a chair on one side of a 5.6 m by 6.4 m room. At the other end, a chair was placed next to a door. The agent, if present, appeared to sit in this chair. See Fig 1 for the room setup. On the back side of the door was one of the four posters showing ten anagrams. The poster was placed such that it was not visible to participants when the door was open, but visible when the door was closed. The placing of the chairs and the posters in the room was chosen specifically to prevent the agent from disappearing completely from the participants’ limited virtual field of view while they were completing the task.

#### Design and procedure

The experiment adopted a 2x2 design, crossing social context and task difficulty. While participants completed an anagram task, social context was manipulated (i.e., the agent was either present or absent), and the task difficulty was either easy or hard. There were four conditions: social-easy, social-hard, alone-easy and, alone-hard. Participants were randomly assigned into one of the 24 possible orderings of the four conditions. Each condition appeared at each of the four serial positions equally across participants, allowing for both a within-subjects analysis over all four trials and a between-subjects analysis only using the participants’ first anagram trials.

**Fig 2:**
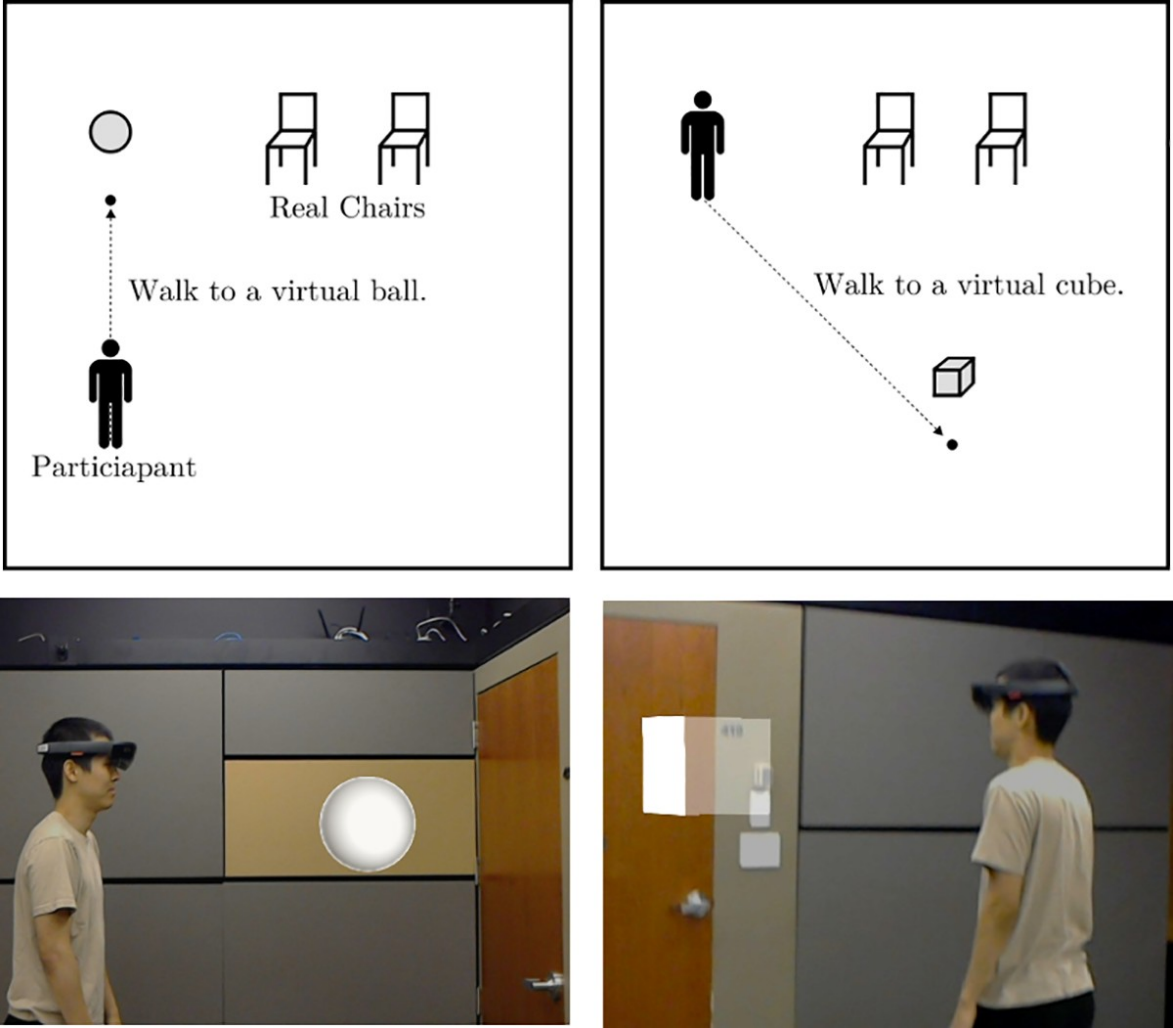
The participant acquainting themselves with the HoloLens by interacting with the sphere and cube.

Between two and five weeks before the date of the experiment, participants filled out a prescreening survey to determine which studies they were eligible for. The prescreening data included participant’s biological sex, which was used to match the agent’s sex to each participant. On the day of the experiment, participants entered the lab and filled out the consent form. Participants were then led to the experiment room, where they completed two short training tasks. First was an anagram-solving task with an example anagram and two training anagrams. This ensured participants could see the anagrams and understood the anagram task. Then, the experimenter fitted the AR headset on the participant. The second task was a simple navigation task involving AR objects. The experimenter asked participants to confirm they saw a virtual ball and then to walk towards that ball until it changed color. This process was repeated with a virtual cube. This second task ensured participants were able to see virtual objects and were comfortable wearing the device. Fig 2 shows this process.

Participants then saw the agent rendered in their virtual field of view, and the experimenter who stood in the room physically. The experimenter stood about a meter to the left of the agent and asked the agent to introduce itself, then the agent provided a short introduction. The text of the introduction is given in S1 Protocol. Depending on condition, the experimenter then informed the participant that the agent would either stay in the room while they completed the task or would leave the room. The experimenter instructed participants to solve as many anagrams as possible in the allotted time, to solve in any order they would like, and to speak the solution word aloud each time they solved an anagram. Then, the experimenter taped the correct poster of anagrams on the back side of the open door, which was not visible to the participant, and instructed the participant to begin solving when the door was closed. The participant’s view of the experiment room is given in Fig 1. The experimenter then waited for exactly 3 minutes outside of the room and returned to the room. This process was repeated three more times, once per combination of social context and difficulty. Participants were then led to a different room where they completed a questionnaire. Once the questionnaire was completed, participants were debriefed.

#### Measures

**Score.** The number of anagrams solved was determined by analyzing the recordings of each of the participants. To expedite analysis, a program was created using Python (version 2.7) to go through the audio and cut large sections of silence for each of the recordings. A human coder then listened to the trimmed recordings and determined which words were spoken by participants. Each participant received a point for each anagram solved correctly. However, a few anagrams had more than one solution. For example, the solution for "RBSCU" was listed as "SCRUB" in Tresselt and Mayzner [50], but multiple participants solved it with the word "CURBS". We included these unintended solutions in the participant’s score, but if a participant said both words (e.g., "CURBS" and "SCRUB") only one solution was counted. Scores ranged from 0 to 10 (*M* = 5.92, *SD* = 3.08).

### Results and discussion

Due to technical issues, six participants were excluded from the analysis. Four participants were excluded because the HoloLens automatically turned off in the middle of the experiment, failing to produce an audio recording. During the session of the other two participants, the network connection between the experimenter's laptop and the HoloLens failed, preventing data collection. After these exclusions, the data from 54 participants (28 female, 26 male) was analyzed.

With proper ordering of conditions we were able to test both a between and within-subject design. While the advantage of a within-subject design is that it accounts for variance due to individual participants, a between-subject has the ability to test initial, novel reactions to a given condition without contamination from previous ones. Thus, we report both analyses separately.

We operationalize social facilitation and inhibition as an interaction effect between the difficulty of the anagram and the presence of the agent on score. For the between-subjects analysis, an ANOVA was performed with the R programming language, version 3.5.1. For the within-subjects analysis, samples were not independent given multiple data points came from the same participant. Consequently, a mixed-effect model, using the ‘nlme’ package version 3.1.137, was used to analyze the within-subjects data. The fixed effects of this model included difficulty, social context, the interaction between difficulty and social context, and condition order. The random effects were the random intercepts per participant.

#### Between-subjects

The statistical assumptions that are necessary for an ANOVA are that residuals are normally distributed and have equal variance among each tested group. The residuals were not different from a normal distribution as determined by a Shapiro-Wilk test (*W* = 0.99, *p* = 0.82). The variances were not significantly different among conditions, (*F*(3, 50) = 0.35, *p* = 0.79).

**Manipulation check.** The main effect of difficulty (easy vs. hard) on score was significant (easy: *M* = 7.68, *SD* = 2.51; hard: *M* = 4.16, *SD* = 2.55; *F*(1, 46) = 63.23, *p* < .001, *d* = 1.95). Participants solved more anagrams in the easy conditions than in the hard conditions indicating that the manipulation of difficulty between conditions was successful.

**Fig 3:**
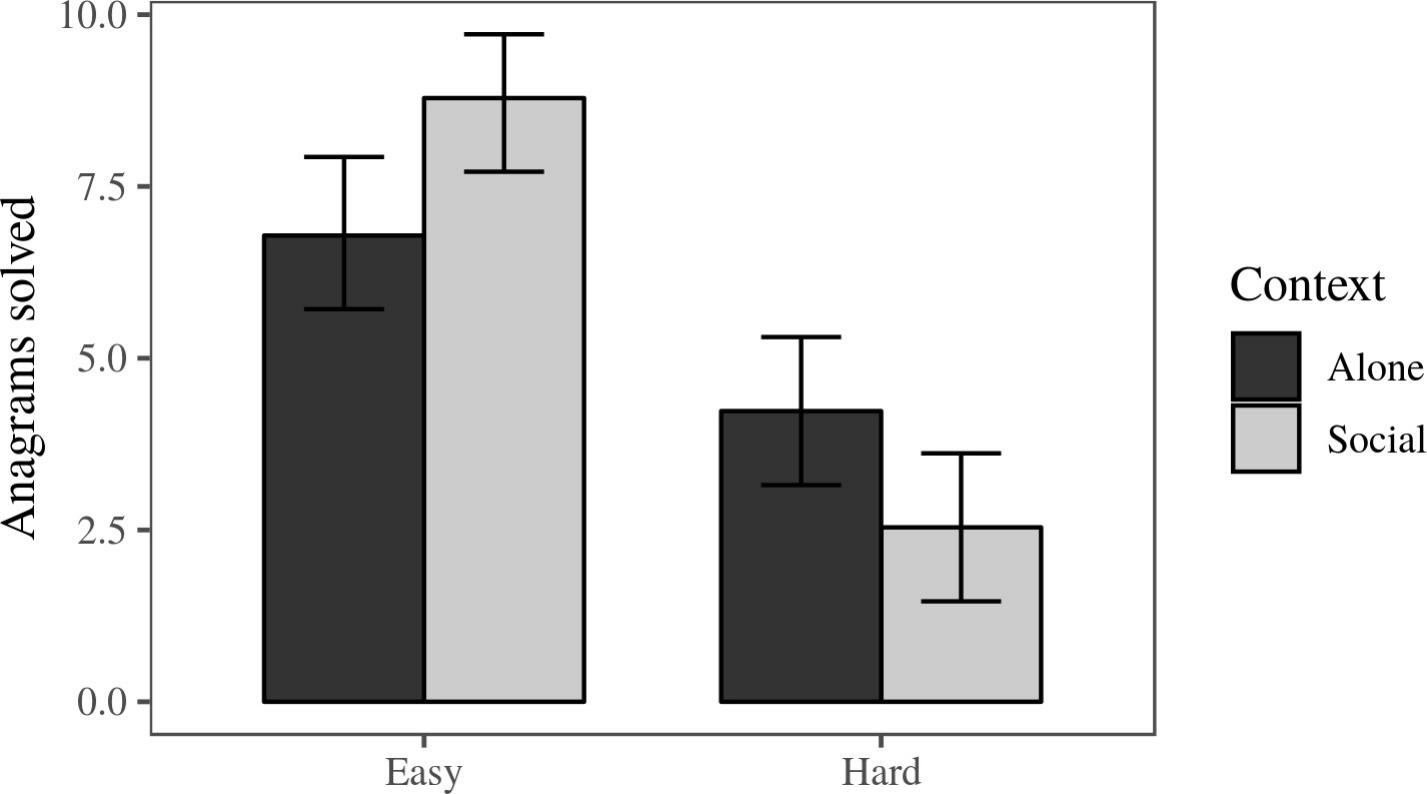
Anagrams solved per condition. This chart displays means and 95% CI’s for the number of anagrams solved in each condition.

**Effect of social context on score.** As expected, the main effect of social context (social vs. alone) on score was not significant (*F* (1, 46) = 0.16, *p* = .69, *d* = 0.07). The interaction effect between social context and anagram difficulty was significant, (*F*(1, 46) = 11.84, *p* < .01, *d* = 0.88). To test the simple effects, we conducted two post-hoc t-tests. Social facilitation predicts that participants will solve more easy anagrams in a social context than when they are alone. This was confirmed, (social: *M* = 8.79, *SD* = 1.97; alone: *M* = 6.79, *SD* = 2.19*; t*(25.71) = 2.54, *p* = 0.03; *d* = 0.96). Social inhibition predicts that participants will solve more hard anagrams alone than socially. This is also confirmed, (social: *M* = 2.54, *SD* = 2.07; alone: *M* = 4.23, *SD* = 2.13*; t*(23.98) = -2.06, *p* = 0.05; *d* = 0.81). Overall, these results confirm Hypothesis 1. Table 1 shows the means and standard deviations, and Fig 3 shows the means and 95% bootstrapped CI’s by condition.

**Table 1:** Means and standard deviations of anagrams solved per condition.

	Social Context			
	Agent		No Agent	
**Task Difficulty**	*M*	*SD*	*M*	*SD*
Easy	8.79	1.97	6.79	2.19
Hard	2.54	2.07	4.23	2.13

*M =* Mean, *SD* = Standard deviation

#### Within-subjects

The statistical assumptions that are necessary for a mixed-effect linear model are that residuals are normally distributed and have equal variance among each tested group. The residuals were not different from a normal distribution as determined by a Shapiro-Wilk test (*W* = 1.00, *p* = 0.79). The variances were not significantly different among groups, whether the groups are defined by our conditions (i.e., social context and difficulty) (*F*(3, 212) = 1.06, *p* = 0.37), participant (*F*(53, 162) = 0.86, *p* = 0.73), or condition serial order (*F*(3, 212) = 0.01, *p* = 1.00).

**Manipulation check.** The main effect of difficulty (easy vs. hard) on score was significant (easy, *M* = 7.68, *SD* = 2.51;. hard, *M* = 4.16, *SD* = 2.55; *b* = -3.42, *t*(158) = -11.66, p < 0.001, *d* = 1.39). Participants solved more anagrams in the easy conditions than in the hard conditions, indicating that the manipulation of difficulty between conditions was successful.

**Effect of social context on score**. The main effect of social context (social vs. alone) on score was not significant (*b* = -0.05, *t*(158) = -0.17, *p* = 0.87, *d* = 0.05). The interaction effect between social context and difficulty was also not significant (*b* = -0.19, *t*(158) = -0.46, *p* = 0.64, *d* = 0.04).

**Condition serial order effects.** The effect of the condition order on score was significant (*b* = 0.19, *t*(158) = 2.03, *p* = 0.04). Participants answered more anagrams over time. These results may be caused by a learning effect and alternatively explain why the repeated-measures analysis did not confirm Hypothesis 1, as change over time dwarfed the effect of condition. Table 2 gives the means and standard deviations of anagram score as well as participant count for each pairing of order and condition.

**Table 2:** Means and standard deviations of anagrams solved per condition and order.

	Conditions (Difficulty / Social Context)			
	Easy / Alone	Easy / Social	Hard / Alone	Hard / Social
Trial	*M (SD)*, *n*	*M (SD)*, *n*	*M (SD)*, *n*	*M (SD)*, *n*
1st	6.79 (2.19), 14	8.79 (1.97), 14	4.23 (2.13), 13	2.54 (2.07), 13
2nd	7.50 (2.75), 12	7.38 (2.50), 13	4.00 (2.67), 15	4.07 (2.20), 14
3rd	8.36 (1.69), 14	7.21 (2.89), 14	4.58 (2.57), 14	4.93 (3.17), 13
4th	8.14 (2.85), 14	7.15 (2.94), 13	4.36 (3.05), 14	4.54 (2.11), 13

Overall, the results from the between-subjects analysis confirm Hypothesis 1 and replicate Park and Catrambone’s [33] social facilitation and inhibition findings within an AR context. These results provide some of the first empirical evidence suggesting that virtual humans in AR can socially influence performance, adding to the literature on the social effects of AR agents [15–18]. However, these effects did not extend to multiple trials. This may be due to the low behavioral realism of the agent, as it does not speak or otherwise act socially except for the short introduction before the first social condition. An alternative explanation is that the participant becomes accustomed to the presence of the agent and so no longer feels social pressure.

## Study 2: Nonverbal behavior

While Study 1 assessed the social influence that virtual humans have on performance, Study 2 examines whether or not users act in accordance with social norms when interacting with an agent in AR. Considering the norms of interpersonal distance, this would mean the subject would not sit directly upon the AR agent, and in regards to eye-contact, participants would rotate bodies to avoid turning their backs on the agent while sitting down. Furthermore, Study 2 examines how the associations formed between an agent’s previously rendered location and the physical space affects social behavior after the agent is no longer visible. Given past research has demonstrated that users tend to follow social norms when interacting with virtual humans [38,39,41], and there is a spatial component associated with AR social interactions, our hypotheses are as follows:

**Hypothesis 2.** *Participants wearing the headset will sit on the chair without the agent more often than on the chair with the agent*.

**Hypothesis 3.** *Participants not wearing the headset will sit on the chair that was empty more often than on the chair where the agent was sitting*.

**Research Question 1.** *Will participants avoid a rotation direction that requires turning their heads away from the agent as they choose a seat in order to maintain eye-contact*?

### Methods

#### Participants

A total of 56 participants (40 female, 16 male) were recruited from Stanford University and the surrounding area. The recruitment and experiment processes were approved by the Stanford IRB under protocol IRB-42052. The participants shown have given written informed consent to publish their likeness under the CC-BY license. Forty-seven of the participants received course credit for participating and 9 were compensated with a $10 Amazon gift card. The mean age of the participants was 21.59 (*SD* = 6.24). To compare with Study 1, we have also binned participants in the same ranges and report that 52 participants were 18–24, 2 participants were 25–35, and 2 participants were 48–65. Given the limited research in this area, we determined our sample size using a heuristic of 30 participants by condition [53]. We planned for a total of 60 participants, but faced difficulties with the technology and fell a few short of that number.

#### Materials

Participants wore the HoloLens. For hardware specifications, see the materials section of Study 1. The virtual content was programmed as an application using Unity version 2017.3.1f1. Vuforia [54] was used to track specific markers in the physical world in order to match the virtual content to specific locations in the experimental room. The position (x, y, z) and orientation (yaw, pitch, roll) data of the headset were collected at 60 Hz. The application was connected to a laptop via a networking component, enabling the experimenter to set up and change the virtual content before and during the experiment.

#### Design and procedure

Participants were randomly assigned into one of two conditions: “Headset” and “Without Headset”. In both conditions, participants wore the headset to begin. In the headset, they saw a virtual agent walk across the room and sit in one of two chairs in front the participant. The chair the agent sat on was randomized for each participant. The agent always matched the biological sex of the participants. After the agent sat on the chair, participants in the Headset condition were asked to sit on one of the two chairs in front of them. In the Without Headset condition, participants were asked to remove the headset once the agent sat down in one of the chairs. Once the headset was removed, participants were asked to sit on one of the two chairs in front of them. To disguise the purpose of our experiment (observing which chair participants chose to sit down), the experimenter simply asked participants to sit on a chair where they would answer a questionnaire that was provided with a clipboard. During the study, the experimenter did not know which chair the agent was sitting on as he could not see what was rendered in the HoloLens. However, inferred awareness of the agent’s position based on participant behavior cannot be ruled out.

The procedure of the experiment began with a set of screening questions. The participant answered the screening questions, which were that the participant did not have any conditions preventing them from using AR (e.g. epilepsy or another seizure disorder), and that they signed the consent form. Then, participants were brought to a 5.6 m by 6.4 m room. The experimenter then introduced the HoloLens and placed it on the participant. Making sure the headset fit properly, the experimenter asked the participant to find a virtual sphere (as described in Fig 2) and to walk toward it. A beeping sound alerted the experimenter that the participant had reached the sphere, at which point the participant was asked to find a virtual cube and walk toward it. When the participant reached the cube, another beeping sound occurred, and a virtual agent appeared. The object-finding task was included to help participants become accustomed to the small virtual field-of-view afforded by the HoloLens.

**Fig 4:**
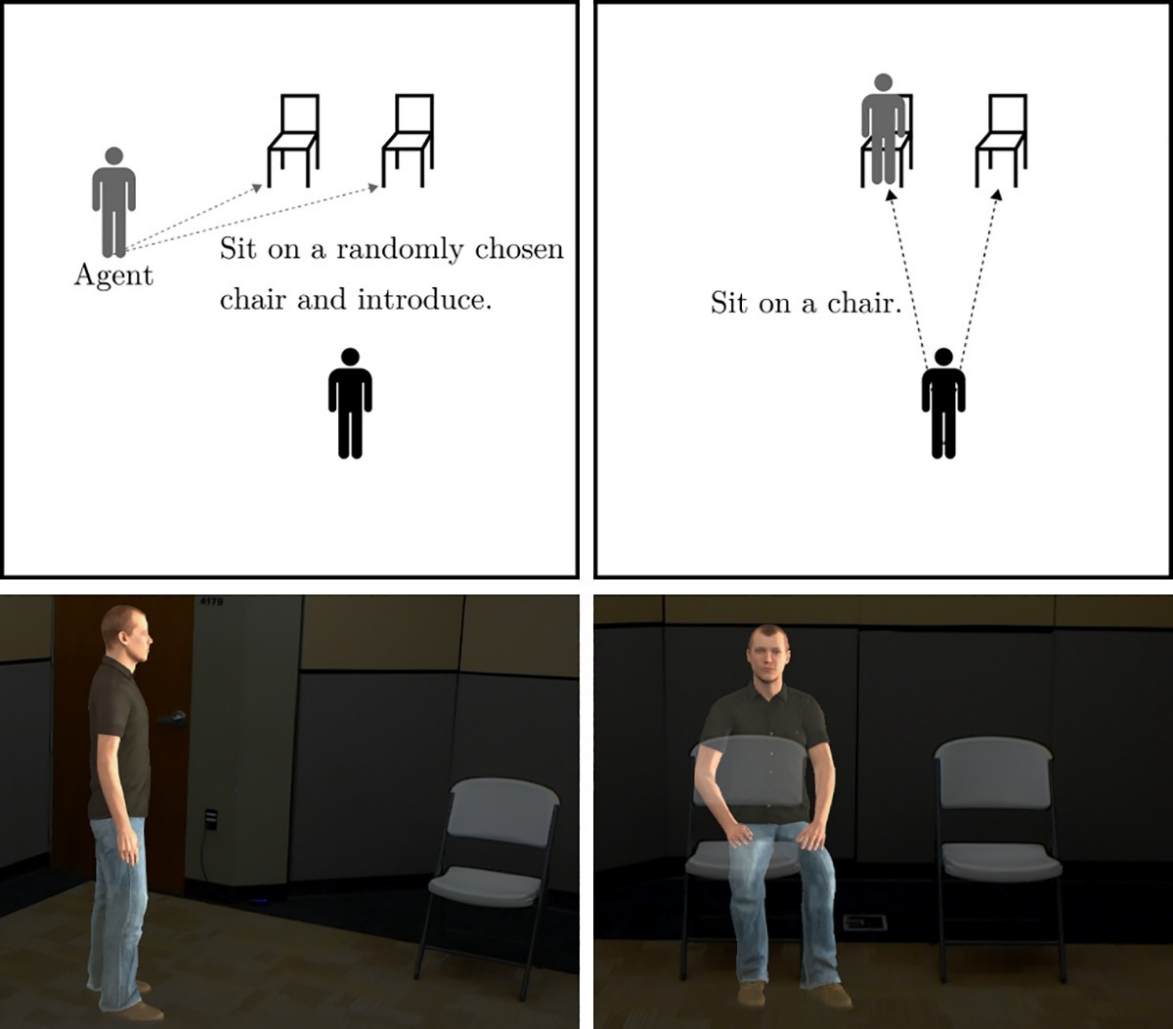
The participant’s interaction with the virtual human. The virtual objects are colored gray.

The agent was then introduced by the experimenter. When the participant looked at the virtual agent, the experimenter triggered the agent to walk and sit on a chair. The agent sat on a randomly chosen chair between two identical chairs placed 1 m apart from each other (Fig 4). After sitting on a chair, the agent directly faced the participant and made an introductory biographical speech for 41 seconds without any additional animations. As with study 1, the participant was not informed of the nature of the virtual person (whether it was an avatar or agent). The content of the speech is listed in S2 Protocol. Once the agent stopped talking, the participant was instructed to either take the headset off and sit on one of the chairs if they were in the Without Headset condition or just to sit on one of the chairs if they were in the Headset condition. Participants in the Headset condition were still able to see the agent sitting on one of the chairs as they sat down.

#### Measures

**Fig 5:**
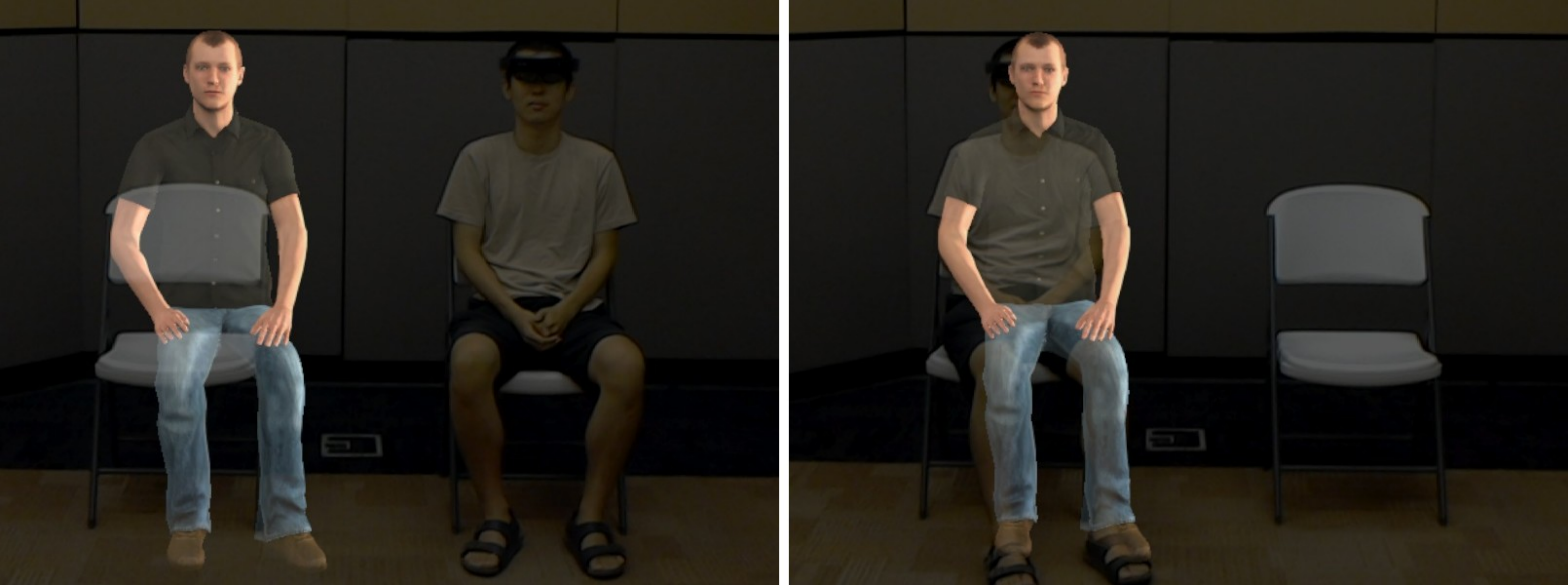
Examples of sitting next to and on the virtual agent.

**Seat choice.** Participants were told to sit on a chair after the virtual agent (sitting on a randomly chosen chair) finished its verbal introduction. The experimenter recorded which chair was chosen by the participant (Fig 5).

**Fig 6:**
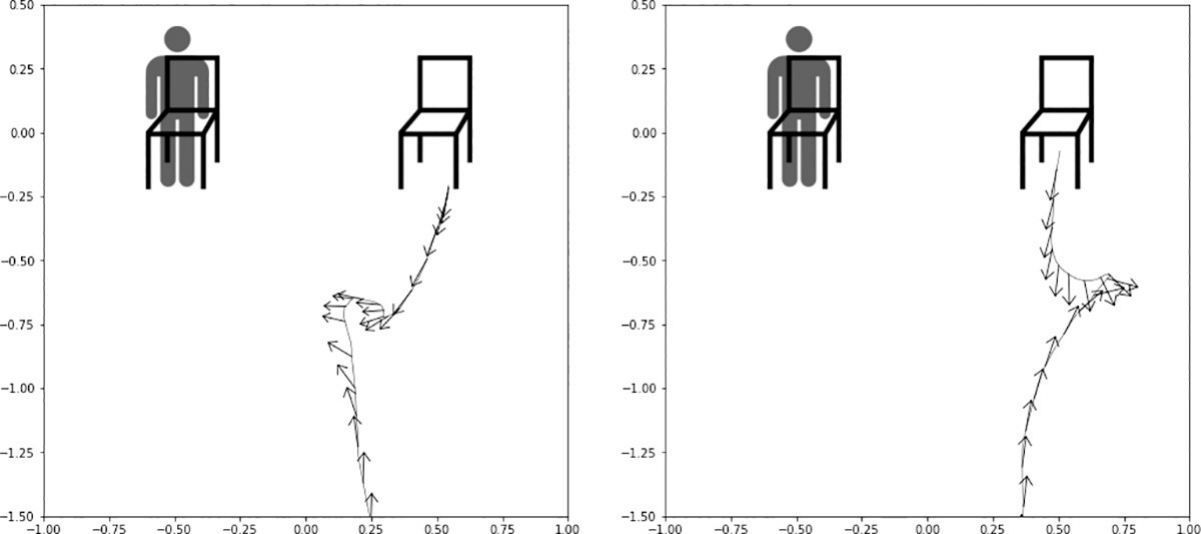
Examples of tracking data visualization of participants. Left panel shows a participant rotating to face the agent (left) and the center panel shows a participant rotating to turn their back to the agent (center). The head orientation (yaw) and head position (x, z) data were collected at 60 Hz. The line depicts the head position along the x-z plane as participants walked, and each arrow depicts the direction on yaw at that position sample.

**Head orientation.** When approaching a chair, there are two directions one can rotate as one turns around and sits down, clockwise or counterclockwise. Using tracking data collected from the headset, we categorized participants based on whether they rotated to face the agent or to turn their back on the agent, which depended on which chair the agent was sitting on. Fig 6 provides examples of how tracking data was visualized for the categorization.

### Results and discussion

**Seat choice.** Table 3 reports the number of observations made on each condition. Hypothesis 2 and 3 were confirmed by a binomial test. The binomial test provides the statistical significance of the deviations of a theoretically expected distribution between two categories of a group. Hypothesis 2 was confirmed since all of the participants with a headset avoided sitting on the agent (*p* < .001). Hypothesis 3 was confirmed as participants who sat without the headset were significantly more likely to sit on the chair without the agent (*p* = .02).

**Table 3:** Number of participants who either sat on or next to the agent in each condition.

	Seat Choice	
Wearing headset when sitting?	On Agent	Next to Agent
Headset	0	27
Without Headset	8	21

**Head Orientation.** Since the head tracking data was collected with the headset, the participants in the Without Headset condition were not included in this analysis. Twenty-five participants out of the twenty-seven participants in the Headset condition rotated in order to face the agent, as opposed to turn their back on it. Assuming that people have an equal chance to turn their heads to the agent or away from the agent while sitting on a chair, results from the binomial test showed that participants were significantly more likely to sit on the chair while turning their heads toward the agent (*p* < .001). This addresses Research Question 1, suggesting that participants will avoid a rotation direction that requires turning their heads away from the agent as they choose a seat in order to maintain eye-contact.

Overall, Study 2 investigated nonverbal behaviors between AR users and agents. Confirming Hypothesis 2, results showed that participants avoided sitting on the chair occupied by the agent and faced the agent while sitting down on the chair next to it. Hypothesis 3 was also confirmed since the majority of participants avoided sitting on the occupied chair even after they took off their headsets.

It is unclear whether the effects observed resulted from the agent’s speech, its appearance, or the mere existence of a three-dimensional digital object in participant’s’ field of view. A limitation of this study is that a participant turning towards the agent when sitting down is equivalent to a participant turning towards the center of the room, and the experiment does not distinguish between the two cases. Furthermore, this experiment demonstrates an effect consistent with social norms, but as designed does provide causal evidence of the agent fully influencing the participant’s nonverbal behavior. Future studies should try to replicate these findings while including both a control condition with no agent present, and a control condition where a non-social object is present on the chair. For example, participants may be less like to sit on a chair with a stack of virtual books, even though the virtual books themselves do not have social influence like an agent does. These control conditions allow further examination of what feature of the agent–its existence or its social ability–accounts for the behavior observed.

## Study 3: Social connectedness

One of the main goals of AR systems is to superimpose virtual content onto the user’s real-world environment with the purpose of providing the user with additional information about their surroundings [2]. This affordance makes it possible for AR users to interact with virtual content that is only visible to them which may make bystanders curious or uncomfortable. Furthermore, virtual content may intentionally or unintentionally be rendered on top of people the AR user is interacting with, potentially disrupting the interaction by causing the violation of social norms or affecting the relationship between users and non-users.

Since the virtual content displayed to an AR user has the potential to occlude their conversation partner, and the presence of see-through AR headsets is likely to disrupt social norms, such as eye-contact, our research questions are as follows:

**Research Question 2.** *Will dyads that interact one of the participants being occluded by virtual content have lower social presence*, *interpersonal attraction*, *and perceived closeness than dyads where no one is occluded by virtual content*?

**Research Question 3.** *In a dyadic face-to-face interaction where one person wears the AR headset and the other does not*, *is there a difference in how participants feel about their respective partner in terms of social presence*, *interpersonal attraction*, *and perceived partner closeness*?

### Method

#### Participants

A total of 102 participants (54 female, 48 male) were recruited from Stanford University. The recruitment and experiment processes were approved by the Stanford IRB under protocol IRB-45030. The participants shown have given written informed consent to publish their likeness under the CC-BY license. Participants were assigned to same-sex dyads in order to account for potential sex effects, creating a total of 51 dyads. Participants were recruited from an undergraduate-level course, however, their ages were not collected during this study. Given the limited research in this area, we determined our sample size using a heuristic of about 30 dyads per condition [53]. We planned to collect 120 participants, but difficulties in recruitment led do a small reduction from our planned sample.

#### Materials

**Fig 7:**
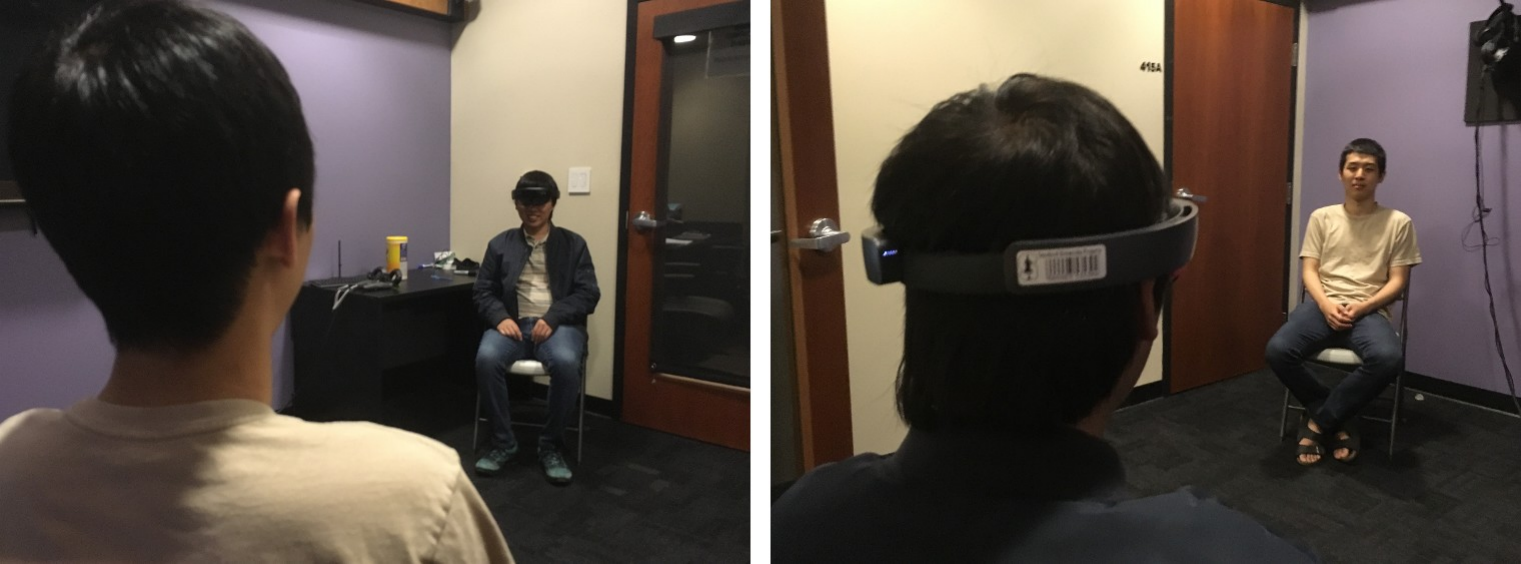
Video shot of a pair of actors simulating the experiment.

Participants who had never interacted with each other met for the first time inside a room (4x4 meters). Within each dyad, one of the participants was randomly selected to wear the HoloLens (for hardware specifications, please see the materials section of Study 1). Participants were seated on two chairs placed in front of each other 2.3 m apart. A video camera equipped with a microphone was stationed in the room to record the dyadic interaction (Fig 7). Participants wearing the HoloLens saw a superimposed university logo that was either placed next to or directly in front of their interaction partner depending on what condition they were randomly assigned to. The entirety of the simulation was programmed using Unity version 2017.3.1.

#### Design and procedure

**Fig 8:**
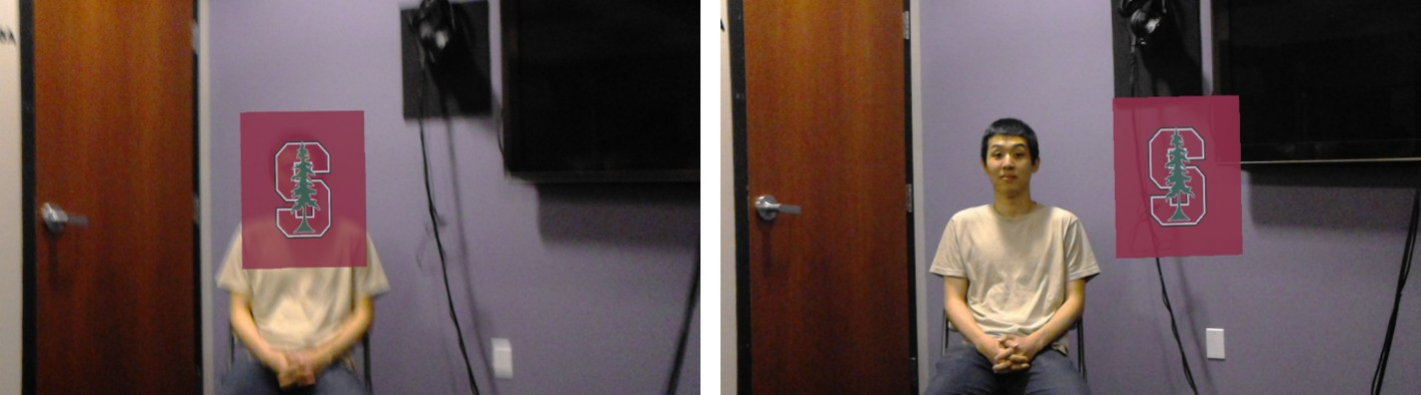
View of the other participant from the AR user’s point of view in the Virtually Occluded condition (left) and Not Virtually Occluded condition (right).

**Fig 9:**
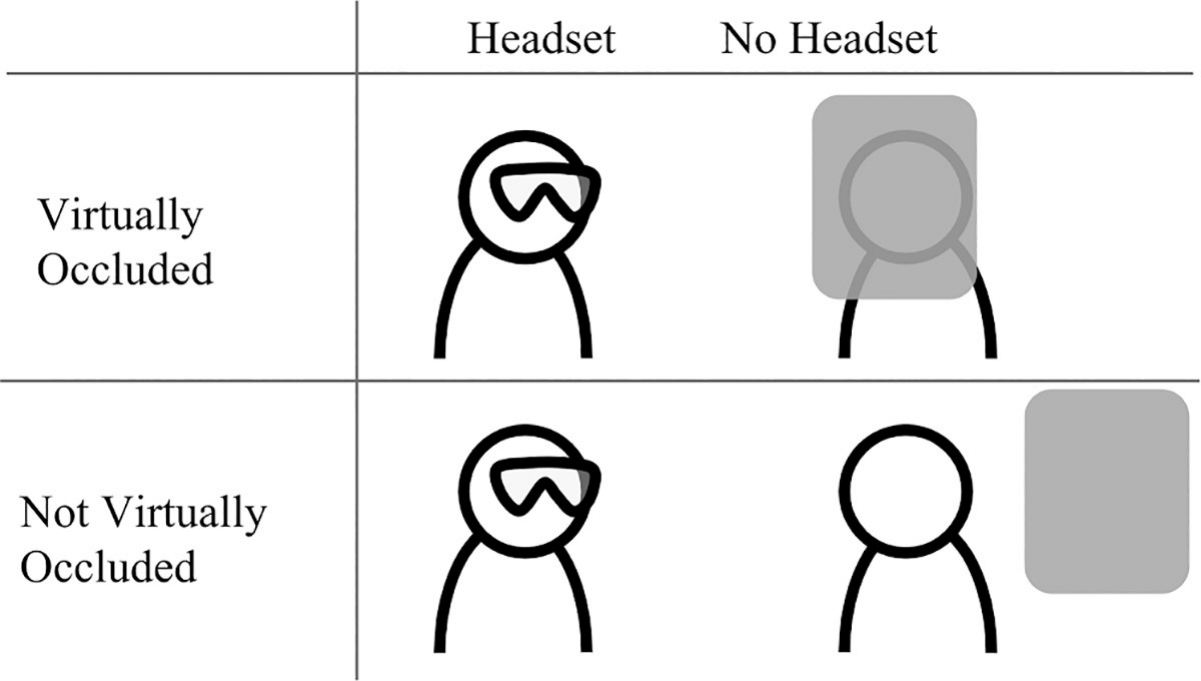
Diagram of conditions for Study 3.

The present study adopted a standard-distinguishable dyadic design in which each dyad was formed of two participants of the same biological sex but each partner differed from each other as part of the experimental manipulation. Participants were randomly selected into one of the two conditions, namely ‘Virtually Occluded’ and ‘Not Virtually Occluded’, and were paired with a participant of their same biological sex. Within each dyad, one of the participants was randomly selected to wear the HoloLens during the interaction while the other participant did not wear an AR headset. In the Virtually Occluded condition, the participant in the dyad wearing the headset saw a university logo occluding their conversation partner’s face (Fig 8, left side). In the Not Virtually Occluded condition, the person wearing the headset saw the same logo next to their conversation partner’s body (Fig 8, right side). The logo did not occlude any part of the conversation partner’s body. In both the Virtually Occluded and Not Virtually Occluded conditions, the conversation partner that did not wear the HoloLens was oblivious to the content their partner was seeing. A pictographic representation of the conditions is provided in Fig 9.

Participants were asked to come to two different locations to prevent them from interacting with each other prior to the study. None of the participants included in the study had met each other before the experiment. After completing the consent form, the participant assigned to wear the headset, regardless of condition, was brought into the lab room, equipped with the HoloLens, and asked to report whether or not they were able to see the university logo. After the participant wearing the HoloLens reported seeing the logo, the second participant was brought into the lab room and asked to sit on the remaining chair.

Once both participants were seated, they were asked to discuss one interesting thing that had happened to them over the past month for 5 minutes. The instructions were adapted from Przybylski and Weinstein [43]. The video recording of the interaction began as soon as the participants started their conversation. The logo displayed on the AR headset disappeared after exactly 5 minutes signaling the end of the interaction. At that moment, experimenters entered the lab room and escorted each participant into two separate rooms where participants completed a questionnaire about their experience. Upon completing the questionnaire, participants were debriefed. S2 Appendix includes all of the measures included in the questionnaire which are described in detail below.

#### Measures

**IOS.** The Inclusion of the Other in the Self (IOS) scale is a single-item, pictorial measure of closeness and connectedness [55]. Pictures are Venn-like diagrams of two circles overlapping, with each circle representing the self and the participant’s conversation partner. The pictures are coded from 1 to 7 with the larger numbers indicating an increasingly closer relationship.

**Social Presence**. Social presence was measured by adapting items from the Networked Minds Social Presence Inventory [56]. Participants were asked to rate their agreement with specific statements on a 5-point Likert-type scale (*1 = Strongly disagree*, *5 = Strongly agree*). Sample items include “I was often aware of my partner in the environment” and “I sometimes pretended to pay attention to my partner”.

**Interpersonal Attraction.** The Interpersonal Attraction scale measures the extent to which participants liked their interaction partner and would enjoy interacting with them in the future [57]. Participants rated their agreement with six statements on a 5-point Likert-scale (*1* = *Strongly disagree*, *5 = Strongly agree*). Sample items include “My partner is the type of person I could become close friends with” and “I like my partner”.

### Results and discussion

Dyadic data violates the assumption of independence needed to perform analysis of variance (ANOVA) [58]. Because of this, a linear mixed model analysis was used to account for the fixed effects condition and headset use (i.e., whether or not there were differences between participants who wore the HoloLens and those who did not), and the random effects of individual interaction partners nested within each dyad, thus adding random slopes and random intercepts to our model. All analyses were carried out in R version 3.5.1 using the “lme4” and “nlme” packages. Table 4 shows the means and standard deviations for each dependent variable by condition.

**Table 4:** Means and standard deviations for all outcome variables by condition and users and non-users.

	Conditions			
	Virtually Occluded		Not Virtually Occluded	
	Headset (n = 25)	No Headset (n = 25)	Headset (n = 26)	No Headset (n = 26)
**Measures**	*M (SD)*	*M (SD)*	*M (SD)*	*M (SD)*
Social Presence	4.22 (.57)	4.52 (.55)	4.49 (.45)	4.45 (.44)
IOS	2.88 (1.5)	3.72 (1.7)	3.73 (1.3)	3.5 (1.4)
Interpersonal Attraction	4.19 (.61)	4.4 (.47)	4.24 (.65)	4.2 (.72)

*M =* Mean, *SD* = Standard deviation

**IOS.** There was no significant difference between the Virtually Occluded and Not Virtually Occluded conditions on IOS scores (*b* = .32, *t* (49) = 1.05, *p* = .30, *d =* .30). However, regardless of condition, participants within each dyad who wore the AR headset during the social interaction reported feeling significantly less connected to their partners than participants who were not wearing the headset (*b* = -.42, *t* (49) = 2.09, *p* = .04, *d =* .59). There was also a marginally significant interaction effect (*b* = .53, *t* (49) = 1.90, *p* = .06, *d* = .54), indicating that when participants wore the HoloLens, those who interacted with a partner that was virtually occluded by the Stanford logo felt marginally less connected to their partner than participants who interacted with a partner that was not virtually occluded (*b* = .85, *t* (49) = 2.17, *p* = .03). However, for participants that did not wear the HoloLens, there was no significant difference in IOS scores regardless of condition (*b* = .22, *t* (49) = .51, *p* = .61).

**Social presence.** There was no significant difference between the Virtually Occluded and Not Virtually Occluded conditions on social presence (*b* = .09, *t* (49) = 0.84, *p* = .40, *d* = .25). Similar to the IOS results, participants within each dyad who wore the AR headset during the social interaction reported feeling significantly less social presence than participants who were not wearing the headset regardless of condition (*b* = .30, *t* (49) = 2.38, *p* = .02, *d* = .68). There was also a marginally significant interaction effect (*b* = .17, *t* (49) = 1.89, *p* = .06, *d* = .54), indicating that when participants wore the HoloLens, those who interacted with a partner that was virtually occluded by the Stanford logo felt marginally less social presence than participants who interacted with a partner that was not virtually occluded (*b* = .26, *t* (49) = 1.81, *p* = .08). However, for participants that did not wear the HoloLens, there was no significant difference in Social Presence scores regardless of condition (*b* = .07, *t* (49) = .50, *p* = .62).

**Interpersonal Attraction.** There was no significant difference between the Virtually Occluded and Not Virtually Occluded conditions on interpersonal attraction (*b* = -.07, *t* (49) = 0.51, *p* = .61, *d* = .15). There was also no significant difference between participants who wore the AR headset during the social interaction and the participants who were not wearing the headset on how much they liked each other regardless of condition (*b* = .10, *t* (49) = 1.44, *p* = .16, *d* = .41). Additionally, there was no significant interaction effect between interpersonal attraction scores and any of the conditions (*b* = .12, *t* (49) = 1.22, *p* = .23, *d* = .35). Study 3 sought to examine the effect that the presence of see-through AR headsets during a dyadic social interaction has on social presence, interpersonal attraction, and perceived partner closeness, and to assess the effect that interacting with someone whose face is either completely occluded by virtual content or not occluded by virtual content at all has on interpersonal outcomes. Analyses showed that individuals within each dyad who wore the AR headset during the social interaction felt less social presence and less connected to their interaction partners who were not wearing an AR headset. Past research has demonstrated that when two people interact with each other but one person actively uses a smartphone during the interaction while the other does not, non-users form significantly more negative impressions of their partners compared to the smartphone users [51]. Our results showed the opposite pattern with AR users reporting lower social presence and IOS scores compared to non-users. It is possible that non-users in our study were not aware that they were being occluded and, therefore, were unaware that they did not have their partner’s full attention. Future studies should experimentally manipulate whether or not non-users are aware that they are being occluded by virtual content to examine whether or not this knowledge affects interpersonal outcomes.

Moreover, results showed that there was no overall significant difference between the two occlusion conditions on social presence, IOS, or interpersonal attraction, suggesting that in this instance, occlusion by virtual content does not significantly affect interpersonal outcomes. However, it is possible that this result was obtained due to our small sample size, the nature of the social interaction task (i.e., talking about something good that had happened to them in the past month for 5 minutes), or the fact that the virtual content displayed in both of our conditions was not interactive. Future studies should try to replicate these findings with a larger sample, different levels of interactivity, and different types of collaborative tasks.

Overall, these results suggest that a social interaction where one of the interactants is occluded by virtual content does not lead to reduced interpersonal attraction, perceived closeness, or social presence when compared to a social interaction where none of the interactants are occluded by virtual content. Additionally, AR users, regardless of condition, tend to have lower social presence and IOS scores compared to their interaction partners who are not wearing an AR headset. However, future studies should try to replicate our findings with more participants in order to gain statistical power.

A limitation of this study is that the virtual logo displayed was not 100% opaque (i.e., the participant’s outline was still visible to their interaction partner wearing the headet) and only covered the participants face (i.e., participant’s mannerisms and body were still visible). Future studies should examine the effect of completely occluding interaction partners with virtual content that does not allow users to see outlines or silhouettes of their interaction partners on interpersonal outcomes. Additionally, given not being able to see your interaction partner resembles other types of mediated communication (e.g. telephone call or one-way skype call where one user does not share their video feed), therefore future studies should try to compare how participant’s behavior or interpersonal outcomes during an AR social interaction differ or resemble other types of mediated communication. Another limitation is the virtual field of view afforded by the AR headset. It is possible that participants adjusted their head so that they could see their partner from their periphery or moved their head so that the virtual content disappeared. Future studies should collect tracking data from both AR users and non-users to identify whether or not nonverbal behavior or social norms (e.g., eye-contact) are affected by interacting with others while using an AR headset.

## General discussion

### Summary of results

The present investigation examined social interactions in AR. In Study 1, a well-known psychological theory (social facilitation/inhibition) was applied to an AR user with a virtual agent. Study 2 investigated whether or not users follow social norms when interacting with virtual humans, and whether or not spatial associations between physical locations and virtual content affect subsequent behavior. Study 3 examined the effect of wearing an AR headset during an interaction with someone who is not wearing a headset and who may or may not be occluded by virtual content.

Study 1 and 2 relate to a question: do AR agents elicit responses similar to real humans? In Study 1, the interaction of task difficulty and social context significantly affected participant performance. Participants solved more easy anagrams and fewer hard anagrams in the presence of an agent than alone, replicating both social facilitation and social inhibition effects. In Study 2, participants acted in accordance with social norms and avoided sitting on the chair occupied by a virtual agent, and a majority of participants did not turn their heads away from the agent while sitting down. The results of these two studies suggest, as Reeves and Nass [59] did with the media equation, that social interactions with agents resemble face-to-face social interactions with humans.

In Study 2 and 3, we examined AR specific situations. Half of the participants of Study 2 were told to sit on a chair after the headset was removed, and they still chose to avoid the seat previously occupied by the agent. In Study 3, we investigated a possible side effect of using an AR headset and having a virtual object rendered on top of an interaction partner within the context of interpersonal communication. As the experimental design implies, we expected virtual objects and the AR device itself to hinder communication as they prevented eye-contact and disrupted common ground between interactants (i.e., one participant in each dyad was able to see virtual content while the other participant did not). Results showed that participants wearing headsets felt significantly less connected to their partners and rated their non-headset partner with significantly lower social presence, even though both interactants were in the same physical room when compared to their partners who were not wearing the headset.

### Limitations and future work

A limitation of this study is that the samples for all three studies were mostly composed of undergraduate students with little demographic variance. Future studies should consider collecting data at different time intervals and manipulating exposure time in AR as an independent variable. Another limitation of these studies is that the realism (behavioral or photographic) was not systematically controlled. Past research has shown that agents exert social influence, and that agency and realism moderate that influence [60]. Future studies should control and examine the effects that different levels of agency and realism in AR have on communication outcomes.

Past research has also investigated participants’ differences in attention between video-recorded and real humans [61,62] and hypothesized one key difference between the two is potential for real-time social interaction. Future work can corroborate this construct in the medium of AR. Some previous work in AR explored some work in joint action, [4–9], which is “any form of social interaction whereby two or more individuals coordinate their actions in space and time to bring about a change in the environment.” Future work should continue this line while being grounded in theories of joint action, task sharing, and action coordination [63–65].

### Implications for theory and AR design

With the media equation, Reeves and Nass [59] suggest that individuals tend to respond to media experiences in very similar ways as real experiences. Given virtual agents are media, specifically designed to be similar to humans, a large portion of our results can be interpreted with their theory. In Study 1 and 2, participants were influenced by agents as they would have been expected to be influenced by humans. More specifically, in Study 1 participants exhibited both social facilitation and inhibition effects while performing tasks in front of an agent the same way that participants in previous research exhibited theses effects in front of real humans. Although, it is important to note that these effects diminished over time. In Study 2, we examined the effect that virtual agents have on participants’ nonverbal behavior and found that participants tend to follow interpersonal distance and eye-contact social norms with agents the way they follow these norms with other humans. However, it is not clear whether this is caused by the agent’s social influence or merely the presence of an interactive three-dimensional object, which was clearly a novel experience for participants.

For designers of AR headsets and applications, Study 1 and 2 provide evidence suggesting that agents exert influence similar to real humans and can affect the way AR users perform tasks in the physical world or move in space (i.e., the positioning of virtual humans within a physical space can affect both where users move to and where they look). When it comes to AR applications, there is a line of research that has focused on the benefit of providing instructions to physical tasks in AR [20–22]. Given the results obtained in Study 1, we suggest applications remove agents or avatars while users perform difficult tasks in order to prevent inhibitions effects. However, when it comes to providing instructions for easy tasks, making the agent or avatar more salient may help AR users improve their performance.

Since AR content tends to be registered in specific physical locations, AR application designers should take into consideration that each user’s physical environment is unique and therefore, each AR user may experience the same virtual content differently depending on the context of their physical environment. Additionally, the rendering of virtual content in specific locations may affect subsequent user behavior or the relationship a user has with a physical space. In Study 2, results showed that even after participants had taken the headset off, they still tended to sit on the chair that was not previously occupied by the agent. While the temporal duration of these effects and whether or not the causal mechanism was social influence is still unknown, these results suggest that AR content may affect how users interact with their physical environment even after they have stopped using the technology, which raises ethical concerns for AR content producers.

Study 3 sheds light on the interpersonal costs associated with using an AR headset during a social interaction with a non-user. The use of AR headsets with non-users seems to hinder social presence and how close users feel to non-users, suggesting that AR headsets may change the quality of social interaction. Whether this hindrance is driven by the AR headset’s mere presence or by the virtual content being displayed is still an open question that should be explored empirically. However, given our limited data, we suggest that applications that could be used while the AR user may be interacting with non-users (e.g., navigation) design their virtual content to be somewhat transparent in order to prevent total occlusion of the non-users.

One thing to consider is how these results generalize to a world in which AR is being used at scale. It is one thing to run set laboratory studies with small numbers of participants, and another issue entirely to predict how this technology will change social interaction when it migrates out into the world. This paper scratches the surface of the social psychological costs and benefits of AR use, but much research is needed to understand the effects of this technology as it scales.

## Supporting information

S1 Appendix Study 1 Anagrams. Anagrams and solution words participants solved.
(DOCX)

S2 Appendix Study 3 Post-Survey. All post-survey measures in Study 3.
(DOCX)

S1 Protocol Study 1 Virtual Human Script. The text spoken by the virtual person to introduce himself or herself in study 1.
(DOCX)

S2 Protocol Study 2 Virtual Human Script. The text spoken by the virtual person to introduce himself or herself in study 2.
(DOCX)

S1 Data Study 1 anagram score data. (CSV)

S2 Data Study 2 tracking and survey data, part 1. (Z01)

S3 Data Study 2 tracking and survey data, part 2. (Z02)

S4 Data Study 2 tracking and survey data, part 3. (Z03)

S5 Data Study 2 tracking and survey data, part 4. (Z04)

S6 Data Study 2 tracking and survey data, part 5. (Z05)

S7 Data Study 3 survey data. (CSV)

## References

[1] Billinghurst M, Clark A, Lee G. A Survey of Augmented Reality. Found Trends Human–Computer Interact. 2015;8: 73–272. 10.1561/1100000049

[2] Azuma RT. A Survey of Augmented Reality. Presence Teleoperators Virtual Environ. 1997;6: 355–385.

[3] Allport GW. The nature of prejudice. Reading, MA: Addison-Wesley; 1954.

[4] Tait M, Billinghurst M. The Effect of View Independence in a Collaborative AR System Comput Support Coop Work. Norwell, MA, USA: Kluwer Academic Publishers; 2015;24: 563–589. 10.1007/s10606-015-9231-8

[5] Wang X, Dunston PS. Comparative Effectiveness of Mixed Reality-Based Virtual Environments in Collaborative Design. IEEE Trans Syst Man Cybern. 2011;41: 284–296.

[6] Kiyokawa K, Billinghurst M, Hayes SE, Gupta A, Sannohe Y, Kato H. Communication Behaviors of Co-Located Users in Collaborative AR Interfaces. Proceedings of the 1st International Symposium on Mixed and Augmented Reality. Washington, DC, USA: IEEE Computer Society; 2002. p. 139—. Available: http://dl.acm.org/citation.cfm?id=850976.854962

[7] Dong S, Behzadan AH, Chen F, Kamat VR. Collaborative visualization of engineering processes using tabletop augmented reality. Adv Eng Softw. 2013;55: 45–55.

[8] Poelman R, Akman O, Lukosch S, Jonker P. As if Being There: Mediated Reality for Crime Scene Investigation. Proceedings of the ACM 2012 Conference on Computer Supported Cooperative Work. New York, NY, USA: ACM; 2012. pp. 1267–1276. 10.1145/2145204.2145394

[9] Lukosch S, Billinghurst M, Alem L, Kiyokawa K. Collaboration in Augmented Reality. Comput Support Coop Work CSCW An Int J. 2015;24: 515–525. 10.1007/s10606-015-9239-0

[10] Asobo Studio. Fragments. Bordeaux, France: Asobo Studio SARL; 2016.

[11] Microsoft. Browse all HoloLens apps [Internet]. 2019 [cited 1 May 2019]. Available: https://www.microsoft.com/en-us/store/collections/hlgettingstarted/hololens

[12] Oh CS, Bailenson JN, Welch GF. A Systematic Review of Social Presence: Definition, Antecedents, and Implications. Front Robot AI. 2018;5: 114 10.3389/frobt.2018.00114 PMC7805699 33500993

[13] Kim K, Billinghurst M, Bruder G, Duh HB-L, Welch GF. Revisiting Trends in Augmented Reality Research: A Review of the 2nd Decade of ISMAR (2008–2017). IEEE Trans Vis Comput Graph. IEEE; 2018; 10.1109/TVCG.2018.2868591 30188833

[14] Steptoe W, Julier S, Steed A. Presence and discernability in conventional and non-photorealistic immersive augmented reality. ISMAR 2014—IEEE International Symposium on Mixed and Augmented Reality—Science and Technology 2014, Proceedings. 2014. pp. 213–218. 10.1109/ISMAR.2014.6948430

[15] Anabuki M, Kakuta H, Yamamoto H, Tamura H. Welbo: An Embodied Conversational Agent Living in Mixed Reality Spaces. Proc CHI’2000, Ext Abstr. 2000; 10–11. 10.1145/633292.633299

[16] Wagner D, Billinghurst M, Schmalstieg D. How Real Should Virtual Characters Be? Proc 2006 ACM SIGCHI Int Conf Adv Comput Entertain Technol. 2006;

[17] Kim K, Bruder G, Welch GF. Blowing in the Wind: Increasing Copresence with a Virtual Human via Airflow Influence in Augmented Reality. In: Bruder G, Cobb S, Yoshimoto S, editors. International Conference on Artificial Reality and Telexistence Eurographics Symposium on Virtual Environments. 2018.

[18] Kim K, Maloney D, Bruder G, Bailenson JN, Welch GF. The effects of virtual human’s spatial and behavioral coherence with physical objects on social presence in AR. Computer Animation and Virtual Worlds. 2017 pp. 1–9. 10.1002/cav.1771

[19] Kim K, Boelling L, Haesler S, Bailenson JN, Bruder G, Welch GF. Does Alexa Need a Body? The Influence of Visual Embodiment and Social Behavior on the Perception of Intelligent Virtual Agents. IEEE International Symposium on Mixed and Augmented Reality. 2018. pp. 1–10.

[20] Henderson SJ, Feiner SK. Augmented reality in the psychomotor phase of a procedural task. 2011 10th IEEE Int Symp Mix Augment Reality, ISMAR 2011. 2011; 191–200. 10.1109/ISMAR.2011.6092386

[21] Marner MR, Irlitti A, Thomas BH. Improving procedural task performance with Augmented Reality annotations. 2013 IEEE Int Symp Mix Augment Reality, ISMAR 2013. IEEE; 2013; 39–48. 10.1109/ISMAR.2013.6671762

[22] Boud AC, Haniff DJ, Baber C, Steiner SJ. Virtual reality and augmented reality as a training tool for assembly tasks. Proc Int Conf Inf Vis. IEEE; 1999;1999–Janua: 32–36. 10.1109/IV.1999.781532

[23] Triplett N. The Dynamogenic Factors in Pacemaking and Competition. Am J Psychol. 1898;9: 507–533.

[24] Allport FH. The influence of the group upon association and thought. J Exp Psychol. 1920;3: 159–182.

[25] Pessin J. The Comparative Effects of Social and Mechanical Stimulation on Memorizing. 1The Am J Psychol. 1933;45: 263–270.

[26] Zajonc RB. Social Facilitation. Science (80-). 1965; 10.1126/science.149.3681.269 14300526

[27] Aiello JR, Douthitt EA. Social facilitation from triplett to electronic performance monitoring. Gr Dyn. 2001;5: 163–180. 10.1037/1089-2699.5.3.163

[28] Bond CF, Titus LJ. Social Facilitation: A Meta-Analysis of 241 Studies. Psychol Bull. 1983;94: 265–292. 10.1037/0033-2909.94.2.265 6356198

[29] Dashiell JF. An experimental analysis of some group effects. J Abnorm Soc Psychol. 1930;25: 190–199.

[30] Hoyt CL, Blascovich J, Swinth KR. Social Inhibition in Immersive Virtual Environments. Presence. 2003;12: 183–195.

[31] Zanbaka CA, Ulinski AC, Goolkasian P, Hodges LF. Effects of Virtual Human Presence on Task Performance. Proc Artif Real Telexistence. 2004; 174–181.

[32] Zanbaka C, Ulinski A, Goolkasian P, Hodges LF. Social Responses to Virtual Humans: Implications for Future Interface Design. Proceedings of the SIGCHI Conference on Human Factors in Computing Systems. 2007. pp. 1561–1570. 10.1145/1240624.1240861

[33] Park S, Catrambone R. Social Facilitation Effects of Virtual Humans. Hum Factors. 2007;49: 1054–1060. 10.1518/001872007X249910 18074704

[34] Ekman P, Friesen W V. The Repertoire of Nonverbal Behavior: Categories, Origins, Usage, and Coding. J Int Assoc Semiot Stud. 1969;1: 49–98.

[35] Hayduk LA. Personal Space: Where We Now Stand. Psychol Bull. American Psychological Association; 1983;94: 293.

[36] Kleinke CL. Gaze and Eye Contact: A Research Review. Psychol Bull. 1986;100: 78–100. 3526377

[37] Blascovich J, Bailenson J. Infinite Reality. 2011.

[38] Slater M, Sadagic A, Usoh M, Schroeder R. {Small-Group} Behavior in a Virtual and Real Environment: A Comparative Study. Presence Teleoperators Virtual Environ. MIT Press; 2000;9: 37–51.

[39] Bailenson JN, Blascovich J, Beall AC, Loomis JM. Equilibrium Theory Revisited: Mutual Gaze and Personal Space in Virtual Environments. Presence Teleoperators Virtual Environ. MIT Press; 2001;10: 583–598.

[40] Bönsch A, Radke S, Overath H, Asché LM, Wendt J, Vierjahn T, et al. Social VR: How Personal Space is Affected by Virtual Agents’ Emotions. 2018 {IEEE} Conference on Virtual Reality and {3D} User Interfaces ({VR}). 2018. pp. 199–206.

[41] Yee N, Bailenson JN, Urbanek M, Chang F, Merget D. The Unbearable Likeness of Being Digital: The Persistence of Nonverbal Social Norms in Online Virtual Environments. CyberPsychology Behav. 2007;10: 115–121. 10.1089/cpb.2006.9984 17305457

[42] Stephenson N. Snow Crash. New York, NY, USA: Bantam Books; 1992.

[43] Przybylski AK, Weinstein N. Can you connect with me now? How the presence of mobile communication technology influences face-to-face conversation quality. J Soc Pers Relat. 2013;30: 237–246. 10.1177/0265407512453827

[44] Misra S, Cheng L, Genevie J, Yuan M. The iPhone Effect: The Quality of In-Person Social Interactions in the Presence of Mobile Devices. Environ Behav. 2016;48: 275–298. 10.1177/0013916514539755

[45] Vanden Abeele MMP, Antheunis ML, Schouten AP. The Effect of Mobile Messaging During a Conversation on Impression Formation and Interaction Quality Comput Hum Behav. Amsterdam, The Netherlands, The Netherlands: Elsevier Science Publishers B. V.; 2016;62: 562–569. 10.1016/j.chb.2016.04.005

[46] Clark HH, Brennan SE. Grounding in communication. Perspect Soc Shar Cogn. 1982; 127–149. 10.1037/10096-006

[47] Due BL. The social construction of a Glasshole: Google Glass and multiactivity in social interaction. PsychNology J. 2015;13: 149–178.

[48] Burgess N, Becker S, King JA, O’Keefe J. Memory for events and their spatial context: models and experiments. Philos Trans R Soc London Ser B. 2001;356: 1493–1503. 10.1098/rstb.2001.0948 11571039 PMC1088531

[49] Cummings JJ, Bailenson JN. How Immersive Is Enough? A Meta-Analysis of the Effect of Immersive Technology on User Presence. Media Psychol. 2016;19: 272–309. 10.1080/15213269.2015.1015740

[50] Tresselt ME, Mayzner MS. Normative solution times for a sample of 134 solution words and 378 associated anagrams. Psychon Monogr Suppl. 1966;1: 293–298.

[51] Kreylos O. On the road for {VR}: Microsoft {HoloLens} at Build 2015, San Francisco Doc-Ok.org. 2015.

[52] Zeller M, Bray B. HoloLens hardware details [Internet]. 2018 [cited 20 Oct 2018]. Available: https://docs.microsoft.com/en-us/windows/mixed-reality/hololens-hardware-details

[53] Hogg R V., Tanis EA. Probability and Statistical Inference. 7th ed Pearson; 2005.

[54] PTC Inc. Vuforia. PTC Inc.; 2017.

[55] Aron A, Aron EN, Smollan D. Inclusion of Other in the Self Scale and the structure of interpersonal closeness. J Pers Soc Psychol. US: American Psychological Association; 1992;63: 596–612. 10.1037/0022-3514.63.4.596

[56] Harms C, Biocca F. Internal Consistency and Reliability of the Networked Minds Measure of Social Presence. 2004;

[57] Davis D, Perkowitz WT. Consequences of responsiveness in dyadic interaction: Effects of probability of response and proportion of content-related responses on interpersonal attraction. J Pers Soc Psychol. US: American Psychological Association; 1979;37: 534–550. 10.1037/0022-3514.37.4.534

[58] McMahon JM, Pouget ER, Tortu S. A guide for multilevel modeling of dyadic data with binary outcomes using SAS PROC NLMIXED. Comput Stat Data Anal. 2006;50: 3663–3680. 10.1016/j.csda.2005.08.008 16926924 PMC1550976

[59] Reeves B, Nass CI. The Media Equation: How people treat computers, television, and new media like real people and places. Chicago, IL, US: Center for the Study of Language and Information; 1996.

[60] Blascovich J. Social influence within immersive virtual environments. Soc Life Avatars. 2002; 127–145. 10.1007/978-1-4471-0277-9_8

[61] Laidlaw KEW, Foulsham T, Kuhn G, Kingstone A. Potential social interactions are important to social attention. Proc Natl Acad Sci. 2011;108: 5548–5553. 10.1073/pnas.1017022108 21436052 PMC3078350

[62] Risko EF, Laidlaw K, Freeth M, Foulsham T, Kingstone A. Social attention with real versus reel stimuli: toward an empirical approach to concerns about ecological validity. Front Hum Neurosci. 2012;6: 1–11. 10.3389/fnhum.2012.00001 22654747 PMC3360477

[63] Sebanz N, Bekkering H, Knoblich G. Joint action: Bodies and minds moving together. Trends in Cognitive Sciences. 2006 pp. 70–76. 10.1016/j.tics.2005.12.009 16406326

[64] Vesper C, Abramova E, Bütepage J, Ciardo F, Crossey B, Effenberg A, et al Joint action: Mental representations, shared information and general mechanisms for coordinating with others. Frontiers in Psychology. 2017 pp. 1–7. 10.3389/fpsyg.2017.00001 28101077 PMC5209366

[65] Wahn B, Kingstone A, König P. Group benefits in joint perceptual tasks—A review. Annals of the New York Academy of Sciences. 2018 pp. 166–178. 10.1111/nyas.13843 29754443

