# Supplementary material for: Social interaction in augmented reality
Source: PLoS One. 2019 May 14;14(5):e0216290. doi: 10.1371/journal.pone.0216290 (PMC6516797; doi:10.1371/journal.pone.0216290)
Supplement: S1 Appendix — Anagrams and solution words participants solved. (DOCX) [file pone.0216290.s001.docx]

**S1 Appendix: Anagrams for Study 1**

The anagrams were selected from Tresselt and Mayzner [1]. First, piloting tests confirmed the median solution times of the anagrams. Then, the experimenter chose twenty easy and twenty hard anagrams from the single-letter solution words table. In order to reduce the time each trial would take, anagrams that took less time were preferred, but in order to reduce individual variance, anagrams of less-common words were passed over.

Anagrams, as listed by poster:

| Group A: Easy | | Group B: Easy | | Group C: Hard | | Group D: Hard | |
| --- | --- | --- | --- | --- | --- | --- | --- |
| ODELM | (MODEL) | NRDKI | (DRINK) | RBSCU | (SCRUB) | EUCNL | (UNCLE) |
| BEAHC | (BEACH) | EGUJD | (JUDGE) | KCLER | (CLERK) | OEPWR | (POWER) |
| MILBC | (CLIMB) | RMCAP | (CRAMP) | IUMCS | (MUSIC) | OAPNR | (APRON) |
| ORLAB | (LABOR) | OHTNM | (MONTH) | DPAOT | (ADOPT) | LRUFO | (FLOUR) |
| UGARS | (SUGAR) | AEWTR | (WATER) | RHTIB | (BIRTH) | SJTUO | (JOUST) |
| EUOHS | (HOUSE) | IFNLG | (FLING) | BNLOE | (NOBLE) | SPEUA | (PAUSE) |
| LCOHT | (CLOTH) | IUEGD | (GUIDE) | PHMNY | (NYMPH) | LTVIA | (VITAL) |
| NTGIA | (GIANT) | ULTFA | (FAULT) | AUGDR | (GUARD) | TAIBH | (HABIT) |
| EOCVI | (VOICE) | NTRAI | (TRAIN) | AEUVL | (VALUE) | HUOCG | (COUGH) |
| AWRLB | (BRAWL) | IHRCA | (CHAIR) | ELCSA | (SCALE) | OCBNA | (BACON) |

**References**

1. Tresselt ME, Mayzner MS. Normative solution times for a sample of 134 solution words and 378 associated anagrams. Psychon Monogr Suppl. 1966;1: 293–298.
