# Supplementary material for: Social interaction in augmented reality
Source: PLoS One. 2019 May 14;14(5):e0216290. doi: 10.1371/journal.pone.0216290 (PMC6516797; doi:10.1371/journal.pone.0216290)
Supplement: S2 Appendix — All post-survey measures in Study 3. (DOCX) [file pone.0216290.s002.docx]

**S2 Appendix: Study 3 Post-experiment Survey**

*Interpersonal Attraction*

How strongly do you agree or disagree with the following statements about your partner?

1. I like my partner

2. I would get along well with my partner.

3. I would enjoy a casual conversation with my partner.

4. My partner is the type of person I could become close friends with.

5. My partner is a good listener.

6. My partner is friendly.

*Inclusion of the Other and the Self*

*
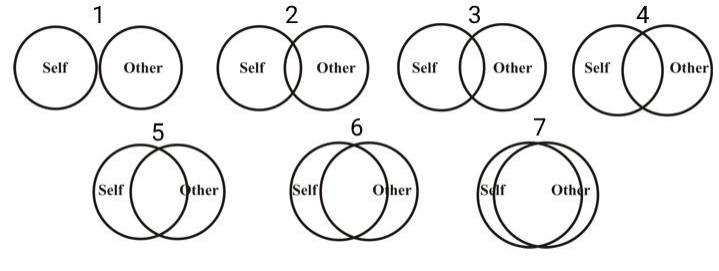
*

1. Please choose which of the figures above most accurately represents your relationship with your conversation partner.

*Social Presence*

1. When you think back on the task, to what extent did you have a sense that you were together with your partner in the same room?

2.  I was often aware of my partner in the environment.

3. I sometimes pretended to pay attention to my partner.

4. My partner sometimes pretended to pay attention to me.

5. My partner paid close attention to me.

6. I paid close attention to my partner.

7. My partner tended to ignore me.

8. I tended to ignore my partner.

9. My partner understood what I meant.

10. I understood what my partner meant.

*Open Ended Responses*

1. Please describe in at least 5 sentences the conversation you had with your partner.

2. Please describe in at least 3 sentences your experience talking with your partner.

3. Please describe in 2-5 sentences the shirt/top that your partner was wearing.

4. What did you think the study was about?

5. Please describe any prior experiences in augmented/mixed reality.
