# Supplementary material for: Social interaction in augmented reality
Source: PLoS One. 2019 May 14;14(5):e0216290. doi: 10.1371/journal.pone.0216290 (PMC6516797; doi:10.1371/journal.pone.0216290)
Supplement: S1 Protocol — The text spoken by the virtual person to introduce himself or herself in study 1. (DOCX) [file pone.0216290.s003.docx]

**S1 Protocol – Study 1 Virtual Human’s introduction**

This script is based on the scripts and identities from a previous study.

“Hi, and welcome to the Virtual Human Interaction Lab. My name is Chris and I'm a research aide in the lab. I'm a junior, originally from Indianapolis, Indiana. In this lab, we study virtual reality. We are in the Department of Communication, and centrally located in the Main Quad. We use state-of-the-art virtual reality headsets, spatialized sound, and advanced haptics to transport you to our virtual worlds. I hope you enjoy your visit today.”
