# Supplementary material for: Social interaction in augmented reality
Source: PLoS One. 2019 May 14;14(5):e0216290. doi: 10.1371/journal.pone.0216290 (PMC6516797; doi:10.1371/journal.pone.0216290)
Supplement: S2 Protocol — The text spoken by the virtual person to introduce himself or herself in study 2. (DOCX) [file pone.0216290.s004.docx]

**S2 Protocol – Study 2 Virtual Human’s introduction**

This script is based on the scripts and identities from a previous study.

“Hi and welcome to the Virtual Human Interaction Lab. My name is Jessica/Michael and I'm a freshman from Denver, Colorado. While I'm currently undeclared, I've been taking music, math, and swimming classes this quarter. I enjoy playing basketball and soccer with friends, cooking and eating anything grilled, and relaxing with Netflix and Hulu. I'm pretty active on Snapchat, Instagram, and Facebook, and I like using Spotify to listen to random playlists. In this lab, we study virtual reality. We are in the Department of Communication, and centrally located in the Main Quad. We use state-of-art virtual reality headsets, spatialized sound, and advanced haptics to transport you to our virtual worlds. I hope you enjoy your visit today.”
